# Congenital anomalies: Case definition and guidelines for data collection, analysis, and presentation of immunization safety data^☆^

**DOI:** 10.1016/j.vaccine.2016.03.047

**Published:** 2016-12-01

**Authors:** Malini DeSilva, Flor M. Munoz, Mark Mcmillan, Alison Tse Kawai, Helen Marshall, Kristine K. Macartney, Jyoti Joshi, Martina Oneko, Annette Elliott Rose, Helen Dolk, Francesco Trotta, Hans Spiegel, Sylvie Tomczyk, Anju Shrestha, Sonali Kochhar, Elyse O. Kharbanda, The Brighton Collaboration Congenital Anomalies Working Group

**Affiliations:** a HealthPartners Institute, United States; b Baylor College of Medicine, Houston, TX, United States; c School of Paediatrics and Reproductive Health, The University of Adelaide, Australia; d Vaccinology and Immunology Research Trials Unit, Women's and Children's Hospital and Robinson Research Institute, University of Adelaide, South Adelaide, Australia; e Department of Population Medicine, Harvard Medical School & Harvard Pilgrim Health, United States; f National Centre for Immunisation Research & Surveillance (NCIRS), Australia; g Discipline of Paediatrics and Child Health, University of Sydney, Australia; h Immunization Technical Support Unit, Public Health Foundation of India, India; i Kenya Medical Research Institute/CDC, Kisumu, Kenya; j Reproductive Care Program of Nova Scotia, Canada; k WHO Collaborating Centre for the Surveillance of Congenital Anomalies, Ulster University, United Kingdom; l Ufficio di Farmacovigilanza, Agenxia Italiana del Farmaco, Italy; m Henry M. Jackson Foundation, United States; n Novartis influenza vaccines, Cambridge, MA, United States; o Sanofi Pasteur, United States; p Global Healthcare Consulting, India

**Keywords:** Congenital anomaly, Birth defect, Congenital disorders, Congenital malformations, Congenital abnormality, Adverse event, Immunization, Guidelines, Case definition

## 1 Preamble

### 1.1 Need for developing case definitions and guidelines for data collection, analysis, and presentation for congenital anomalies as an adverse event following immunization

Congenital anomalies, also commonly referred to as birth defects, congenital disorders, congenital malformations, or congenital abnormalities, are conditions of prenatal origin that are present at birth, potentially impacting an infant's health, development and/or survival. We will use the term congenital anomalies in this report. Congenital anomalies encompass a wide array of structural and functional abnormalities that can occur in isolation (i.e., single defect) or as a group of defects (i.e., multiple defects). Multiple defects may occur as part of well-described associations, such as the non-random co-occurrence of Vertebral anomalies, Anal atresia, Cardiac defects, Tracheoesophageal fistula, and/or Esophageal atresia, Renal and Radial anomalies, and Limb defects (VACTERL) [1].

Congenital anomalies vary substantially in severity. Some congenital anomalies are associated with spontaneous abortion, stillbirth, or death in the early postnatal period. Global deaths due to congenital anomalies decreased from 750.6 thousand in 1990 to 632.1 thousand in 2013, with respective age-standardized death rates of 11.0 and 8.7 per 100,000 [2]. Subtypes of fatal congenital anomalies (with estimated number of global deaths in 2013 in thousands) are congenital heart anomalies (323.4), neural tube defects (68.9), Down's syndrome (36.4), and chromosomal unbalanced rearrangements (17.3) [2]. Other congenital anomalies may have little impact on survival. Anomalies which affect an infant's life expectancy, health status, physical or social functioning may be described as “major” anomalies. In contrast, “minor” anomalies are those with little or no impact on health or short-term or long-term function [3]. We have chosen to focus on major anomalies for this case definition due to their impact on public health and pre-existing structure for surveillance and reporting by large national and international organizations.

The causes of congenital anomalies are wide-ranging, with many anomalies remaining of undetermined etiology. Structural anomalies are often due to errors in embryogenesis occurring at critical periods of fetal development. Critical exposure periods during pregnancy can vary by organ system or type of anomaly. However, first trimester (gestational age 1–13 weeks) is generally considered the highest risk period. Medications, infectious agents, and environmental toxins have all been implicated as teratogens; illicit drugs and other maternal exposures can also disrupt fetal development and increase the risk for one or more congenital abnormalities [1]. Some structural and many functional defects are attributed to underlying genetic defects or chromosomal abnormalities. These defects may be due to one or both parents being genetic carriers, one or both parents sharing the disease state, or the occurrence of de novo mutations [4]. The timing of clinical recognition of major anomalies varies both by type of defect and by access to health care.

To date, multiple studies have investigated congenital anomaly outcomes following maternal vaccination, for both recommended and inadvertent vaccination.

#### 1.1.1 Vaccinations routinely recommended during pregnancy

##### 1.1.1.1 Influenza vaccine, including seasonal and pandemic vaccines

Many countries routinely recommend that pregnant women receive influenza vaccine at any time during pregnancy [5], [6]. Thus, studies evaluating the potential for these vaccines to impact embryogenesis or risks for congenital anomalies are of critical importance. Maternal immunization during pregnancy with inactivated influenza vaccine is associated with a brief increase in maternal inflammatory biomarkers [7], [8]. At the time of publication, there was no data to support an association between the maternal inflammatory response to vaccination and fetal development and risk for congenital anomalies.

As of March 2014, congenital anomaly data from more than 4000 pregnant women who received different types of adjuvanted and non-adjuvanted influenza vaccination during the first trimester, and over 19,000 during any trimester, were published [9], [10], [11], [12], [13], [14], [15], [16], [17], [18], [19], [20] and comprehensively reviewed [21]. Of individual studies, the largest that included first trimester exposures reported pregnancy outcomes for 323 woman immunized with adjuvanted or non-adjuvanted A(H1N1)v2009 influenza vaccines and 1329 control subjects. The rate of major malformations did not vary between the two cohorts (all trimesters: OR 0.87; 95% confidence interval [CI] 0.38, 1.77; preconception and first trimester exposure: OR 0.79; 95% CI 0.13, 2.64) [16]. The review authors concluded that maternal influenza vaccination is not associated with an increased risk of congenital malformations. However, statistical imprecision, and clinical and methodological heterogeneity of included studies made it impossible to totally exclude harm [21]. A 2014 Cochrane systematic review combining five studies in a meta-analysis also found influenza immunization during pregnancy was not associated with a higher risk of congenital anomalies, pooled estimate OR 1.06 (95% CI, 0.90, 1.25) [11], [12], [13], [16], [17], [22].

Since March 2014 there have been at least three retrospective studies published investigating congenital anomaly outcomes following monovalent influenza A (H1N1) vaccines [23], [24], [25]. The largest of the three studies was conducted in Lombardy, Italy, during the pandemic period (October 1, 2009–September 30, 2010) and included 6246 pregnant women immunized with a MF59 adjuvanted pandemic A (H1N1) vaccine [24]. Pregnancies were excluded if either chromosomal aberrations or congenital viral infections were reported in the birth registry. Cases were identified with ICD-9 coding and retained according to EUROCAT guidelines. Unmatched analysis identified 284/6246 (4.5%) cases of congenital malformations in the immunized cohort and 3246/79,925 (4.1%) in the unimmunized cohort, OR 1.13 (95% CI, 0.99, 1.28), and propensity matched OR 1.14 (95% CI, 0.99, 1.31) [24]. Rates and estimates were also available for specific anomalies.

##### 1.1.1.2 Tetanus diphtheria, acellular pertussis vaccines (Tdap)

Many countries recommend administration of the acellular pertussis vaccine during the third trimester of pregnancy [26], [27]. One placebo randomized controlled trial, conducted from 2008 to 2012, examined infant congenital anomaly outcomes following maternal Tdap administration during pregnancy. Between 30 and 32 weeks gestation, 33 women received the Tdap vaccine and 15 received a placebo vaccine, with crossover immunization postpartum. In the vaccinated cohort one infant had a congenital anomaly, as compared to two infants with congenital anomalies in the control group [28]. To date, two retrospective observational studies of Tdap administration during pregnancy have been published in the United States; both suggest there is not a significantly increased risk of major congenital anomalies in infants born to mothers who were vaccinated during pregnancy [29], [30]. The remaining evidence regarding the safety of pertussis containing vaccines is derived from passive surveillance [31].

Maternal and neonatal tetanus remain problematic in geographic areas where childbirth occurs under conditions that do not meet minimum standards of hygiene and immunization coverage of the population is low. In these regions, women with inadequate immunization history are recommended to receive two doses of tetanus toxoid (TT) containing vaccine as early as possible during pregnancy [32]. Between 1959 and 1965 a large prospective study in the United States was conducted that included 337 mother and child pairs evaluated for TT vaccine exposure before 20 weeks gestation. The authors estimated a standardized relative risk (SRR) of 1.19 (95% CI, 0.70, 1.87) for any anomaly following tetanus vaccination during pregnancy [33]. In more recent case-control studies, increased risks of congenital anomalies following maternal TT vaccination were not observed [34], [35]. In one of these studies, 30 women (55% of vaccinated women) were exposed during the first three gestational months [34]. In the other, timing of vaccination during pregnancy was not reported [35].

#### 1.1.2 Vaccinations inadvertently administered during pregnancy

Many vaccines are routinely administered to women of reproductive age, increasing the opportunities for inadvertent vaccination during pregnancy. Thus, continued monitoring of birth outcomes among women inadvertently exposed during pregnancy remains a priority.

##### 1.1.2.1 Live virus vaccines (rubella, measles, mumps, oral poliovirus, yellow fever, and varicella)

Inadvertent maternal vaccination with a live virus vaccine is associated with replication of the vaccine virus and likely a more robust maternal inflammatory response than that observed for inactivated vaccines. However, of greater concern, maternal infections with rubella, varicella, cytomegalovirus and other viruses, during critical periods of fetal development, are associated with specific groupings of congenital anomalies or syndromes [36]. Thus, there is a theoretical risk for inadvertent maternal vaccination with a live virus vaccine to result in a fetal infection and subsequently increase risk for one or more congenital anomalies.

A review of the inadvertent administration of live vaccines (monovalent or combined rubella, oral poliomyelitis virus, and yellow fever vaccines) to pregnant females suggests no evidence of adverse pregnancy outcomes [37]. The incidence of congenital rubella syndrome (CRS) following inadvertent rubella vaccination of pregnant women has been evaluated in several countries in Europe, the United States, Canada, Iran and Latin America. Among more than 3500 susceptible women inadvertently vaccinated against rubella shortly before or in the first trimester of pregnancy, no cases of CRS were reported [38], [39], [40], [41], [42], [43], [44], [45].

No specific studies have been conducted on pregnancy outcomes following inadvertent measles or mumps vaccination. Passive surveillance of vaccine exposures prior to conception and during pregnancy has not indicated an increased risk of congenital malformation or spontaneous abortion [46], but there is not sufficient information to exclude such a risk.

Oral poliovirus vaccine (OPV), containing live attenuated poliovirus types 1, 2, and 3, has been widely used since the 1960s to protect pregnant women and neonates against poliomyelitis. The possible development of viremia following immunization and a few cases suggestive of vaccine-associated anomalies including an unexplained report about fatal spinal cord neuronal damage following maternal immunization in an immune mother have been documented [47]. However, no population-based controlled studies are available to confirm the significance of these individual reports. In response to a poliovirus epidemic in Finland during 1985, OPV was given to 94% of the entire population, including pregnant women. There was no observed increase in the rates of growth retardation, perinatal deaths, prematurity or congenital anomalies in the infants exposed to OPV in utero in comparison with the expected rates [48].

Yellow fever vaccination has been documented in several hundred pregnant women. The risks of adverse outcome of pregnancy and childbirth appear to be similar to those in the general population [49], [50].

Data from a U.S. registry of pregnant women who inadvertently received varicella vaccine either 3 months before, or at any time during, pregnancy, showed that, among the 587 prospectively enrolled women (including 131 live births to women known to be varicella-zoster virus-seronegative), there was no evidence of congenital varicella syndrome [51]. The rate of occurrence of congenital anomalies from prospective reports in the registry was similar to reported rates in the general U.S. population (3.2%) and the anomalies showed no specific pattern or target organ.

##### 1.1.2.2 Human papillomavirus (HPV) vaccine

To date, data on the safety of the bivalent HPV (2vHPV), 4-Valent HPV (4vHPV), and 9-Valent HPV (9vHPV) vaccines administered inadvertently during pregnancy are based on the pre-licensure clinical studies and post-marketing surveillance studies. The latter have been mostly passive, voluntary reporting registry surveillance, which extended for the 4vHPV vaccine until 2012 and is ongoing for the 9vHPV vaccine since licensure by the US FDA in 2015. As of today, there are no data to suggest an increased risk of congenital malformations following exposure to HPV vaccines during pregnancy, but overall numbers of cases have been low, potentially limiting statistical power and precluding the ability to definitively rule out associations of the HPV vaccines with specific anomalies.

A pooled analysis of 42 pre-licensure clinical studies of the 2vHPV vaccine that included 479 pregnancies in which date of last menstrual period occurred between 30 days prior to 45 days after vaccination did not find an increased risk of congenital anomalies, when compared to 414 controls with similar timing of vaccination (1.7 vs. 2.2%) [52]. Similarly, a pooled analysis of five pre-licensure clinical studies of the 4vHPV vaccine did not find a significant difference in the rate of congenital anomalies when comparing 2008 pregnant women receiving vaccination with 2029 pregnant women receiving placebo (2.0 vs. 1.5%, *p* = 0.20) [53].

Post-marketing data on the safety of HPV vaccines administered during pregnancy include manufacturer-sponsored registries of the 2vHPV and 4vHPV vaccines, reports on the 4vHPV vaccine to the Vaccine Adverse Event Reporting System (VAERS), a passive surveillance system in the United States, and an observational cohort study of the 2vHPV vaccine conducted using data from the United Kingdom. Voluntary reporting is known for underreporting of congenital anomalies if not detected at birth, and over-reporting bias for anomalies is common with the use of retrospective reports. The manufacturer-supported registries have therefore only used prospective inadvertent vaccine exposure reports for the calculation of the congenital anomaly rates [54]. The manufacturer-sponsored registries observed overall rates of major congenital malformations that were consistent with the background rates in the populations [5 of 189 live born infants (2.6%) and 37 of 1527 live born infants (2.4%), respectively in the 2vHPV and 4vHPV registries] [55], [56]. Additionally, in an analysis of 4vHPV vaccine reports submitted between June 2006 and December 2013 to VAERS, only two major congenital malformations were reported out of all infants born to 147 women who received 4vHPV during pregnancy [57]. Published data from a formal epidemiologic study on the safety of HPV vaccines during pregnancy in the post-licensure setting come from an observational cohort study in the Clinical Practice Research Datalink; no difference was observed in the percentage of congenital malformations resulting from pregnancies in which pregnancy initiation occurred between 30 days prior to 45 days after 2vHPV vaccination (7 of 119 pregnancies, 5.9%) vs. 120 days to 18 months after vaccination (23 of 350 pregnancies, 6.6%) [58]. Nakalembe et al., conducted a systematic review of 14 studies that evaluated vaccine safety following administration of 2vHPV and 4vHPV vaccines in low and middle income countries [58]. Of the 14 studies, four included information related to pregnancy outcomes; no difference was found between groups in these 4 studies.

Results from the multi-national, double-blind, randomized phase IIb/III trial of the 9vHPV in 14,215 women have recently been published. Pregnancy was reported in 1192 participants in the 9vHPV group and 1129 participants in the 4vHPV group [59]. Pregnancy outcome data was available for 85% of pregnancies. Congenital anomalies were reported in a total of 32 infants and 9 fetuses and rates did not differ between groups (20 in the 9vHPV group and 21 in the 4vHPV group). Among pregnancies with an estimated date of conception within 30 days prior to or after 4vHPV or 9vHPV vaccine administration, (representing 8% of pregnancies with known outcome), no congenital anomalies were reported [59].

##### 1.1.2.3 Meningococcal vaccine

Evidence on the safety of administration of meningococcal vaccination during pregnancy is scarce. A systematic review conducted in 2012 identified 6 studies evaluating the safety of Meningococcal Polysaccharide Vaccine (MPSV) in pregnancy [60]. None of the included studies suggested any adverse outcomes, including birth defects, for infants born to mothers who received MPSV during pregnancy. However, the total study population included 335 women which may be too small to evaluate rare outcomes such as congenital anomalies.

The safety information on meningococcal conjugate vaccines (MCV) is derived from passive surveillance [61], [62]. These data do not suggest harmful events on birth outcomes, including congenital anomalies, when MCV is administrated to pregnant women. To date, no data are available on the safety of new Meningococcal B vaccines when administered during pregnancy.

#### 1.1.3 Background summary

The evidence on potential risks for congenital anomalies following maternal immunization is mostly reassuring. However, studies to date have been limited by insufficient sample sizes, varied definitions for outcomes, and use of non-biologically feasible exposure windows. One or more congenital anomalies are estimated to occur in 3% of pregnancies [63]. However, specific isolated defects generally occur at rates of 1 per 10,000 to 1 per 100,000 births [64]. Given the variability in types and causes of birth defects, future studies of maternal vaccine safety will need much larger sample sizes or alternative approaches (e.g., case-control studies) to detect risks for congenital anomalies, both isolated defects and groups of defects.

#### 1.1.4 Established definitions

Subtle differences exist among the various definitions for congenital anomalies used by organizations specializing in congenital anomalies and development. The National Institute of Child Health and Human Development (NICHD), Metropolitan Atlanta Congenital Defects Program (MACDP), National Birth Defects Prevention Network (NBDPN), and the March of Dimes use the term “birth defects” to describe congenital anomalies. NBDPN subcategorizes birth defects into major and minor anomalies. The definitions used by these organizations focus on both structural and functional abnormalities that are present at birth that have significant health consequences. Please see Table 1 for a complete list of these organizations and the definitions used.

There is no uniformly accepted definition of congenital anomalies, or more specifically, of congenital anomalies following maternal immunization. This is a missed opportunity, as data comparability across trials or surveillance systems would improve data interpretation and promote the scientific understanding of the event. Through the provision of standardized case definitions and guidelines, this document is intended to improve reliability and comparability of data collected from immunized patients and controls in clinical trials, as well as provide a framework for consistently monitoring the safety of vaccines currently recommended during pregnancy or available to women of reproductive age. Such data can be used in the assessment of whether or to what extent a vaccine administered during pregnancy may increase a woman's risk for having a live birth or fetal demise with one or more congenital anomalies. The case definitions and guidelines are intended to be applicable in diverse geographic, administrative, and cultural regions, adaptable to both high and low resource settings.

### 1.2 Methods for the development of the case definition and guidelines for data collection, analysis, and presentation for congenital anomalies as an adverse events following immunization

Following the process described in the overview paper [65] as well as on the Brighton Collaboration Website (http://www.brightoncollaboration.org/internet/en/index/process.html), the Brighton Collaboration *Congenital Anomalies Working Group* was formed in 2015 and included members with backgrounds in clinical medicine, research, public health and industry. The composition of the working and reference group as well as results of the web-based survey completed by the reference group with subsequent discussions in the working group can be viewed at: http://www.brightoncollaboration.org/internet/en/index/working_groups.html.

To guide the decision-making for the case definition and guidelines, a literature search was performed using Medline and Embase, including the terms Pregnant Women/OR Pregnancy/OR pregnant.ti,ab. OR pregnancy.ti,ab. AND Vaccines/OR Vaccination/OR (inadvertent ADJ3 vaccin*).ti,ab. OR (vaccin* ADJ3 pregnan*).ti,ab. AND exp congenital abnormalities/OR birth defect*.ti,ab. OR congenital abnormalit*.ti,ab. OR congenital malformation*.ti,ab. OR (risk ADJ2 (f?etus OR infant* OR bab* OR biolog*)).ti,ab. The search was limited to English language articles and resulted in the identification of >400 references. All abstracts were screened for possible reports of congenital anomalies following immunization. Over 60 articles with potentially relevant material were reviewed in more detail, in order to identify studies using case definitions or, in their absence, providing clinical descriptions of the case material. This review resulted in a detailed summary of >60 articles, including information on the study type, the vaccine, the diagnostic criteria or case definition put forth, the time interval since time of immunization, and any other symptoms. Multiple general medical, pediatric and infectious disease text books were also searched.

The literature search yielded publications in which terminology was inconsistent. An inventory comprising 6 relevant case definitions (Table 1) of congenital anomalies was made available to working group members.

### 1.3 Rationale for selected decisions about the case definition of congenital anomalies as an adverse event following immunization

The group of conditions comprising “Congenital Anomalies” is quite diverse. Our workgroup suggests that isolated congenital anomalies can be stratified into three broad categories:

1. *External structural defects* (e.g., cleft lip or gastroschisis)
2. *Internal structural defects* (e.g., congenital cardiac defects or intestinal atresias)
3. *Functional defects* (e.g., galactosemia or Gaucher's disease).

As previously mentioned, congenital anomalies might occur in isolation (single defect) or as a group of defects (multiple defects) which are often part of well-described associations (e.g., VACTERL). In addition, there may be overlap between categories for certain congenital anomalies. For example, chromosomal defects are often associated with major internal structural defects.

For external structural, internal structural and functional defects, the timing of clinical recognition varies by defect type and by access to health care. For example, in high resource settings, many structural congenital anomalies are diagnosed prenatally through ultrasound or other advanced imaging. Similarly, in high resource settings, functional anomalies may be diagnosed through genetic screening following amniocentesis, chorionic villi sampling, or maternal blood testing. If not detected prenatally, including in low resource settings, external structural defects are usually evident at the time of birth. In contrast, it is common in both high and low resource settings for both internal structural defects and functional defects to be diagnosed in the days, weeks, or months following birth. The diverse range of congenital anomalies and their varied clinical presentation highlights the difficulty of assigning a single classification system. In addition, major congenital anomalies may result in spontaneous abortion, stillbirth or an elective therapeutic abortion, and therefore may not be captured if data collection is limited to live births. It is therefore emphasized, that the possibility of congenital anomalies should always be considered when evaluating etiology for a spontaneous abortion or stillbirth.

Within the definition context, we have assigned four levels of diagnostic certainty to each category listed above. For each category, a fifth level is included to indicate that the event does not meet the case definition for congenital anomalies. The case definition has been formulated such that the Level One definition is highly specific for the condition. As maximum specificity normally implies a loss of sensitivity, additional diagnostic levels have been included in the definition, offering a stepwise increase of sensitivity from Level One down to Level Three, while retaining an acceptable level of specificity at all levels. In this way it is hoped that all possible cases of congenital anomalies can be captured. The diagnostic levels must not be misunderstood as reflecting different grades of clinical severity. They instead reflect diagnostic certainty for the presence of a particular congenital anomaly.

When evaluating the possibility of a congenital anomaly, conditions and syndromes due to either known maternal conditions (e.g., pre-eclampsia or gestational diabetes) or prematurity (e.g., patent foramen ovale or patent ductus arteriosus) should be distinguished from standalone congenital anomalies not related to known causes.

It needs to be re-emphasized that the grading of definition levels is entirely about diagnostic certainty, not clinical severity of an event. Thus, clinically severe anomalies may appropriately be classified as Level Two or Three rather than Level One if there is no evidence of a specific confirmatory test. However, detailed information about the severity of the event should always be recorded, as specified by the data collection guidelines.

The meaning of “Sudden Onset” and “Rapid progression” in the context of congenital anomalies is not applicable as the anomaly or predisposition to develop the anomaly is, by definition, present at birth.

We have attempted to provide adequate diagnostic specificity without being overly restrictive in order to create definitions that are applicable in both high and low resource settings. With regards to specific pathology, radiology, or laboratory findings necessary to meet the case definition, these findings will vary based on the specific congenital anomaly being evaluated. The current definitions refer to broad categories of anomalies; more comprehensive definitions for specific congenital anomalies, (e.g., Tetralogy of Fallot) including well-defined associations or combinations of anomalies (e.g., CHARGE) have been developed elsewhere and are beyond the scope of this working group [3]. We have included appendices of specific major congenital anomalies listed by groups across the globe conducting birth defects surveillance. In addition, our classification is specific to major anomalies, affecting survival, physical or social functioning. Classification of minor anomalies, occurring as isolated or multiple defects, is beyond the scope of this paper.

A treatment response or its failure, is not in itself diagnostic, and may depend on variables like clinical status, time to treatment, and other clinical parameters. For congenital anomalies, treatment is often a surgical intervention. In the absence of standard pathology, radiology, or laboratory findings, documentation of specific treatments (e.g., surgical reports) may be used in evaluating case status. Our definitions account for the possibility of an early surgical correction or, for functional anomalies, a definitive treatment (e.g., stem cell transplant or dietary restrictions).

#### 1.3.1 Timing of vaccination during pregnancy

It is widely recognized that first trimester is the most critical period for teratogen exposure during pregnancy with regards to subsequent effects on fetal development [66]. However, there is variability in the timing of fetal development by gestational week [67]. In addition, more precise timing of embryogenesis depends on the organ or anomaly of interest. With this in mind, the timing of maternal vaccine exposure needs to be both biologically plausible and consistent among studies in order for associations between congenital anomalies and maternal vaccination to provide reliable information. In order to allow for wider windows of teratogen exposure, account for potential errors in assigning a date of conception and gestational age, and focus on exposures during the most plausible time period for development of congenital anomalies, we recommend that maternal vaccination from 30 days prior to conception to 20 weeks gestational age be included in the case definition. We understand that some will choose to include maternal vaccination outside this time period. However, if a true risk during the biologically plausible exposure window exists, inclusion of exposures in periods outside that window may bias results toward the null and make comparison among studies difficult. Regardless of what exposure time period is chosen, the time interval between maternal immunization and diagnosis of a congenital anomaly should be recorded to provide additional information when evaluating the association between maternal vaccination and congenital anomalies. The clinical manifestations and time-lines are dependent on the congenital anomaly being evaluated.

We postulate that a definition designed to be a suitable tool for testing causal relationships requires ascertainment of the outcome (e.g., congenital anomalies) independent from the exposure (e.g., immunisations). Therefore, to avoid selection bias, a restrictive time interval from maternal immunization to diagnosis of congenital anomalies should not be an integral part of such a definition. Instead, where feasible, details of this interval should be assessed and reported as described in the data collection guidelines.

Further, congenital anomalies often occur outside the controlled setting of a clinical trial or hospital. In some settings it may be impossible to obtain a clear gestational age at vaccination, particularly in less developed or rural settings. In order to avoid selecting against such cases, the Brighton Collaboration case definition avoids setting arbitrary time frames.

It is important to differentiate congenital anomalies with well-documented causes from congenital anomalies without clear etiologies. Therefore, while the case definition includes congenital anomalies which are part of well-known syndromes associated with maternal medications (e.g., anti-epileptic medications), toxins (e.g., alcohol), and infections (e.g., rubella), we recommend altering the analysis plan if a study includes these cases [66], [68]. For example, a congenital heart defect associated with fetal alcohol syndrome would be included in a study of congenital anomalies following immunization. However, the analysis plan would differentiate between this congenital anomaly and others which are not due to a known alternative etiology. Similarly, congenital anomalies associated with prematurity will be included during data collection, even if the congenital anomaly is clearly attributable to prematurity, and then accounted for during data analysis.

#### 1.3.2 Guidelines for data collection, analysis and presentation

As mentioned in the overview paper, the case definition is accompanied by guidelines which are structured according to the steps of conducting a clinical trial, i.e. data collection, analysis and presentation. Neither case definition nor guidelines are intended to guide or establish criteria for management of ill infants, children, or adults. Both were developed to improve data comparability. As many studies of congenital anomalies following immunization will occur as part of post-marketing surveillance, it is our hope that the following definitions can also be applied in observational studies.

### 1.4 Periodic review

Similar to all Brighton Collaboration case definitions and guidelines, review of the definition with its guidelines is planned on a regular basis (i.e. every three to five years) or more often if needed.

## 2 Case definition of major congenital anomalies

### 2.1 For all levels of diagnostic certainty

A major congenital anomaly is a structural or functional defect with the following three characteristics:

1. Of prenatal origin
2. Present at the time of live birth or fetal demise, or in utero
3. Affecting (or has the propensity to affect) the health, survival, or physical or cognitive functioning of the individual

The majority of structural congenital anomalies are diagnosed before 2 years of age, usually within the first 6 months of life, and are defined by:

**Major External Structural Defects**
  Level 1 of diagnostic certainty for major external structural defects*
    • Alterations in external anatomy visible at the time of live birth and persistent beyond the immediate peripartum period unless surgically repaired
      OR
    • Alterations in external anatomy visible in a stillbirth or in the products of conception of a spontaneous or therapeutic abortion
      AND
    • Confirmed by documentation of a diagnosis made by a clinician experienced in diagnosing congenital anomalies and with the highest level of morphology training for the specific setting**
**Level 2 of diagnostic certainty for major external structural defects**
  • Alterations in external anatomy visible at the time of live birth and persistent beyond the immediate peripartum period unless surgically repaired
    OR
  • Alterations in external anatomy visible in a stillbirth or in the products of conception of a spontaneous or therapeutic abortion
    AND
  • Confirmed by documentation of a diagnosis made by a clinician with some experience diagnosing congenital anomalies***
**Level 3 of diagnostic certainty for major external structural defects**
  • Alterations in external anatomy visible at the time of live birth and persistent beyond the immediate peripartum period unless surgically repaired
    OR
  • Alterations in external anatomy visible in a stillbirth or in the products of conception of a spontaneous or therapeutic abortion****
    AND
  • Confirmed by documentation of a diagnosis made by a trained maternal or child health care provider with at least minimal experience diagnosing congenital anomalies
    OR
  • For live births, confirmed using individual (ICD-9/ICD-10) codes or as part of an ICD-9/ICD-10 code based algorithm, where the outcome (individual code or algorithm) has been validated*****
**Level 4 of diagnostic certainty (insufficient evidence to confirm) for major external structural defects**
  • Alterations in external anatomy visible at the time of live birth and persistent beyond the immediate peripartum period unless surgically repaired
    OR
  • Alterations in external anatomy visible in a stillbirth or in the products of conception of a spontaneous or therapeutic abortion
    AND
  • Confirmed by medical record review******
    OR
  • Confirmed in claims data (ICD-9/ICD-10 diagnoses)******
    *Please see Appendix A for a list of major external structural defects
    **In high resource settings with access to subspecialists, such as the United States, these clinicians could be geneticists, neonatologists, pathologists, or other relevant subspecialists while in low and middle income countries, diagnosis by a general physician trained in morphology could be sufficient
    ***In high resource settings, such as the United States, these clinicians might include physicians, nurse practitioners, or physician's assistants trained in pediatrics, obstetrics, or family medicine while in low and middle income countries, diagnosis by a general physician trained in morphology could be sufficient
    ****In cases of therapeutic or spontaneous abortion, if products of conception cannot be used for detailed morphologic exam, detection of an external structural defect on prenatal ultrasound could be classified as Level 3
    *****Validation should be conducted in the same data source, with clinical diagnosis or chart review as the gold standard and a positive predictive value of ≥80%
    ******Where there is insufficient detail in the chart or lack of validation in outcome to meet criteria for Level 3
**Major Internal Structural Defects**
  Level 1 of diagnostic certainty for major internal structural defects*
    • Alterations in internal anatomy present at the time of live birth** and persistent beyond the immediate peripartum period unless surgically repaired
      AND
    • Confirmed by definitive imaging study*** or intraoperative diagnosis
      OR
    • Alterations in internal anatomy detected during autopsy for a stillbirth, spontaneous or therapeutic abortion confirmed by documentation by a pathologist or other relevant subspecialist
  **Level 2 of diagnostic certainty for major internal structural defects**
    • Alterations in internal anatomy present at the time of live birth** and persistent beyond the immediate peripartum period unless surgically repaired
      AND
    • Confirmed by documentation of a diagnosis made by a clinician experienced in diagnosing congenital anomalies and with the highest level of morphology training for the specific setting**** without definitive imaging or intraoperative evaluation
      OR
    • For stillbirth, spontaneous or therapeutic abortion, internal structural defect is visible by ultrasound or other imaging modality prenatally
  **Level 3 of diagnostic certainty for major internal structural defects**
    • Alterations in internal anatomy present at the time of live birth** and persistent beyond the immediate peripartum period unless surgically repaired
      AND
    • Confirmed by documentation of a diagnosis made by a clinician with some experience diagnosing congenital anomalies*****
      OR
    • Confirmed using individual (ICD-9/ICD-10) codes or as part of an ICD-9/ICD-10 code based algorithm, where the outcome (individual code or algorithm) has been validated******
  **Level 4 of diagnostic certainty (insufficient evidence to confirm) for major internal structural defects**
    • Alterations in internal anatomy present at the time of live birth** and persistent beyond the immediate peripartum period unless surgically repaired
      OR
    • Alterations in internal anatomy present at time of stillbirth, spontaneous abortion, or induced abortion
      AND
    • Confirmed through medical record review, with the medical record demonstrating that the anomaly was present at the time of live birth or time of fetal demise, and that the anomaly was diagnosed by a trained maternal or child health care provider with minimal experience diagnosing congenital anomalies
      OR
    • Confirmed by claims data (ICD-9/ICD-10 diagnoses)*******
      *Please see Appendix B for a list of major internal structural defects
      **For pyloric stenosis, the propensity to develop this condition is present at birth
      ***Type of definitive imaging study depends on the specific anomaly
      ****In high resource settings with access to subspecialists, such as the United States, these clinicians could be geneticists, neonatologists, pathologists, or other relevant subspecialists while in low and middle income countries, diagnosis by a general physician trained in morphology could be sufficient
      *****In high resource settings such as the United States, these clinicians might include physicians, nurse practitioners, or physician's assistants trained in pediatrics, obstetrics, or family medicine while in low and middle income countries, diagnosis by a general physician trained in morphology could be sufficient
      ******Validation should be conducted in the same data source, with clinical diagnosis or chart review as the gold standard and a positive predictive value of ≥80%
      *******Where there is insufficient detail in the chart or lack of validation in outcome to meet criteria for Level 3
      Additionally, many functional congenital anomalies are diagnosed before 2 years of age, usually within the first 6 months of life, but some functional defects may not be diagnosed until later in life and are defined by:
**Functional Defects**
  Level 1 of diagnostic certainty for major functional defects*
    • For live births, alterations in functioning of one or more organs or body parts not due to a structural defect, present at the time of birth (or propensity to develop alteration present at live birth), and persistent beyond the immediate peripartum period, unless treated through gene therapy or stem cell transplantation
      OR
    • For stillbirths, spontaneous or therapeutic abortions, alterations in functioning of one or more organs or body parts, not due to a structural defect
      AND
    • Confirmed by definitive diagnostic study**
  Level 2 of diagnostic certainty for major functional defects*
    • For live births, alterations in functioning of one or more organs or body parts not due to a structural defect, present at live birth (or propensity to develop alteration present at live birth), and persistent beyond the immediate peripartum period, unless treated through gene therapy or stem cell transplantation
      OR
    • For stillbirths, spontaneous or therapeutic abortions, alterations in functioning of one or more organs or body parts, not due to a structural defect
      AND
    • Confirmed by documentation of a diagnosis made by a clinician experienced in diagnosing congenital anomalies and with the highest level of training in the diagnosis of functional defects for the specific setting***
  Level 3 of diagnostic certainty for major functional defects*
    • For live births, alterations in functioning of one or more organs or body parts not due to a structural defect, present at live birth (or propensity to develop alteration present at live birth), and persistent beyond the immediate peripartum period, unless treated through gene therapy or stem cell transplantation
      OR
    • For stillbirths, spontaneous or therapeutic abortions, alterations in functioning of one or more organs or body parts, not due to a structural defect
      AND
    • Confirmed by documentation of a diagnosis made by a clinician with some experience diagnosing functional defects****
      OR
    • Confirmed using individual (ICD-9/ICD-10) codes or as part of an ICD-9/ICD-10 code based algorithm, where the outcome (individual code or algorithm) has been validated*****
  Level 4 of diagnostic certainty (insufficient evidence to confirm) for major functional defects*
    • For live births, alterations in functioning of one or more organs or body parts not due to a structural defect, present at the time of live birth (or propensity to develop alteration present at live birth), and persistent beyond the immediate peripartum period, unless treated through gene therapy or stem cell transplantation
      OR
    • For stillbirths, spontaneous or therapeutic abortions, alterations in functioning of one or more organs or body parts, not due to a structural defect
      AND
    • Confirmed through medical record review, with the medical record demonstrating that the anomaly was present at the time of live birth or time of fetal demise, and that the anomaly was diagnosed by a trained maternal or child health care provider who is not a qualified geneticist, neonatologist, pathologist, subspecialist, pediatrician, obstetrician, or family medicine practitioner
      OR
    • Confirmed by claims data (ICD-9/ICD-10 diagnoses)******
      *Please see Appendix C for a list of major functional defects
      **Type of confirmatory study (e.g., chromosome analysis, FISH) depends on the specific anomaly
      ***In high resource settings with access to subspecialists, such as the United States, these clinicians could be geneticists, neonatologists, pathologists, or other relevant subspecialists while in low and middle income countries, diagnosis by a general physician trained in morphology could be sufficient
      ****In high resource settings, such as the United States, these clinicians might include physicians, nurse practitioners, or physician's assistants trained in pediatrics, obstetrics, or family medicine while in low and middle income countries, diagnosis by a general physician with some training in diagnosis of functional defects could be sufficient
      *****Validation should be conducted in the same data source, with clinical diagnosis or chart review as the gold standard and a positive predictive value of ≥80%
      ******Where there is insufficient detail in the chart or lack of validation in outcome to meet criteria for Level 3

## 3 Guidelines for data collection, analysis and presentation of congenital anomalies

It was the consensus of the Brighton Collaboration *Congenital Anomalies Working Group* to recommend the following guidelines to enable meaningful and standardized collection, analysis, and presentation of information about congenital anomalies. However, implementation of all guidelines might not be possible in all settings. The availability of information may vary depending upon resources, geographical region, and whether the source of information is a prospective clinical trial, a post-marketing surveillance or epidemiological study, or an individual report of congenital anomalies. Also, as explained in more detail in the overview paper in this volume, these guidelines have been developed by this working group for guidance only, and are not to be considered a mandatory requirement for data collection, analysis, or presentation.

### 3.1 Data collection

These guidelines represent a desirable standard for the collection of data on availability following immunization to allow for comparability of data, and are recommended as an addition to data collected for the specific study question and setting. The guidelines are not intended to guide the primary reporting of congenital anomalies to a surveillance system or study monitor. Investigators developing a data collection tool based on these data collection guidelines also need to refer to the criteria in the case definition, which are not repeated in these guidelines.

Guidelines numbered below have been developed to address data elements for the collection of adverse event information as specified in general drug safety guidelines by the International Conference on Harmonization of Technical Requirements for Registration of Pharmaceuticals for Human Use [69] and the form for reporting of drug adverse events by the Council for International Organizations of Medical Sciences [70]. These data elements include an identifiable reporter and patient, one or more prior maternal immunisations, and a detailed description of the adverse event, in this case, of congenital anomalies following maternal immunization. The additional guidelines have been developed as guidance for the collection of additional information to allow for a more comprehensive understanding of congenital anomalies following maternal immunization.

#### 3.1.1 Source of information/reporter

For all cases and/or all study participants, as appropriate, the following information should be recorded:

(1) Date of report.
(2) Name and contact information of person reporting^2^ and/or diagnosing the congenital anomaly(ies) as specified by country-specific data protection law.
(3) Name and contact information of the investigator responsible for the subject, as applicable.
(4) Relation to the patient (e.g., immunizer [clinician, nurse], family member [indicate relationship], other).

#### 3.1.2 Vaccinee/control

##### 3.1.2.1 Demographics

For all cases and/or all study participants, as appropriate, the following information should be recorded:

(5) Case/study participant identifiers (e.g., first name initial followed by last name initial) or code (or in accordance with country-specific data protection laws).
(6) Date of birth, stillbirth, or spontaneous or therapeutic abortion, if applicable estimated gestational age at time of fetal demise, age of mother, age of infant or gestational age of fetus, race and ethnicity of both infant and mother, and sex of fetus.
(7) For infants: Gestational age and birth weight.

##### 3.1.2.2 Clinical and immunization history

For all cases and/or all study participants, as appropriate, the following information should be recorded:

(8) For the mother, pre-conception medical history, including hospitalizations, underlying diseases/disorders, and medications as well as medical history during pregnancy such as exposure to substances related to major congenital anomalies, tobacco use, alcohol use, illicit drug use, pre-immunization signs and symptoms including identification of indicators for, or the absence of, a history of allergy to vaccines, vaccine components or medications. Specific focus should be on maternal medical conditions associated with increased risk for having an infant with a congenital anomaly (e.g., diabetes).
(9) Also, for the mother, any medication history (other than treatment for the event described) prior to, during, and after immunization including prescription and non-prescription medication with a specific focus on potentially teratogenic medication exposures. Use of prenatal vitamins and folic acid should also be noted.
(10) Maternal immunization history (i.e. previous immunizations and any adverse event following immunization (AEFI)), in particular occurrence of a congenital anomaly in a prior pregnancy following previous maternal immunization.

#### 3.1.3 Details of the immunization

For all cases and/or all study participants, as appropriate, the following information should be recorded:

(11) Date and time of maternal immunization(s).
(12) Description of vaccine(s) (name of vaccine, manufacturer, lot number, dose (e.g., 0.25 mL, 0.5 mL, etc.) and number of dose if part of a series of immunisations against the same disease). The composition and volume of the diluent used as well as information about whether the diluent was from the same or a separate container should also be recorded.
(13) The anatomical sites (including left or right side) of all immunisations (e.g., vaccine A in proximal left lateral thigh, vaccine B in left deltoid).
(14) Route and method of administration (e.g., intramuscular, intradermal, subcutaneous, and needle-free (including type and size), other injection devices).
(15) Needle length and gauge.

#### 3.1.4 The adverse event

(16) For all cases at any level of diagnostic certainty and for reported events with insufficient evidence, the criteria fulfilled to meet the case definition should be recorded.
  Specifically document:
(17) Clinical description of signs and symptoms of one or more congenital anomalies, and if there was medical confirmation of the event (e.g., patient seen by physician).
(18) Date/time of first observation^3^ of congenital anomaly and diagnostic confirmation^4^ and final outcome.^5^
(19) Concurrent signs, symptoms, and diseases.
(20) Measurement/testing – relevant laboratory testing, imaging results, surgical and pathologic reports.
(21) Treatment given for congenital anomaly(ies), especially whether surgical intervention was required.
(22) Physical and developmental outcome^5^ at last observation for living infants.
(23) Objective clinical evidence supporting classification of the congenital anomaly as “major,” varies depending on congenital anomaly evaluating.
(24) Exposures other than maternal immunization during pregnancy (e.g., maternal medications, environmental) considered potentially relevant to the reported event.

#### 3.1.5 Miscellaneous/general

(25) The duration of surveillance for congenital anomalies includes any anomalies identified after the date of vaccination.
(26) The duration of follow-up reported during the surveillance period should be predefined likewise. Although most congenital anomalies will be clinically recognized in the first 30 days of life, some may not be evident until 12–24 months or later.
(27) Methods of data collection should be consistent within and between study groups, if applicable.
(28) Follow-up of cases should attempt to verify and complete the information collected as outlined in data collection guidelines 1 to 24.
(29) Investigators of patients with congenital anomalies should provide guidance to reporters to optimize the quality and completeness of information provided.
(30) Reports of congenital anomalies should be collected throughout the study period regardless of the time elapsed between immunization and the adverse event. If this is not feasible due to the study design, the study periods during which safety data are being collected should be clearly defined.

### 3.2 Data analysis

The following guidelines represent a desirable standard for analysis of data on congenital anomalies to allow for comparability of data, and are recommended as an addition to data analyzed for the specific study question and setting.

(31) Reported events should be classified in one of the following five categories including the four levels of diagnostic certainty. Events that meet the case definition should be classified according to the levels of diagnostic certainty as specified in the case definition. Events that do not meet the case definition should be classified in the additional categories for analysis.

**Event classification in 5 categories**^6^
**Event meets case definition**
  Level 1: *Criteria as specified in the Major Congenital Anomalies case definition*
    *Specify Major External Structural, Major Internal Structural or Major Functional*
  Level 2: *Criteria as specified in the Major Congenital Anomalies case definition*
    *Specify Major External Structural, Major Internal Structural or Major Functional*
  Level 3: *Criteria as specified in the Major Congenital Anomalies case definition*
    *Specify Major External Structural, Major Internal Structural or Major Functional*
**Insufficient Evidence to Confirm case definition**
  Level 4: *Criteria as specified in the Major Congenital Anomalies case definition*
    *Specify Major External Structural, Major Internal Structural or Major Functional*
**Event does not meet case definition**
  Level 5: *Not a major congenital anomaly*

In addition, major congenital anomalies attributed to an alternative cause (e.g., congenital CMV) should still be recorded and identified as likely attributable to a known cause.

(32) The interval between maternal immunization and reported congenital anomaly could be defined as the date/time of immunization (with regards to gestational age) to the date/time of clinical recognition^7^ of the first symptoms and/or signs consistent with the definition. If few cases are reported, the concrete time course could be analyzed for each; for a large number of cases, data can be analyzed in the following increments:

**Time Interval**

Days prior to mother's last menstrual period
0 to <14 weeks gestational age (highest risk for congenital anomaly)
14 to <20 weeks gestational age (lower risk for congenital anomaly)
≥20 weeks gestation (unlikely to be in risk window for congenital anomaly)

Periods of infancy for age of clinical recognition of congenital anomaly.

**Table:** 

Time period	Days
Prenatal	<Day 1 of life
Neonatal^a^	1–27^b^
Early neonatal^a^	1–6^b^
Late neonatal^a^	7–27
Post neonatal	28–364

a Use either Neonatal or divide into early neonatal and late neonatal.

b Day 1 = first 24 h of life.

(33) If more than one measurement of a particular criterion is taken and recorded, the value corresponding to the greatest magnitude of the adverse experience could be used as the basis for analysis. Analysis may also include other characteristics like qualitative patterns of criteria defining the event.
(34) The distribution of data (as numerator and denominator data) could be analyzed in predefined increments (e.g., measured values, times), where applicable. Increments specified above should be used. When only a small number of cases are presented, the respective values or time course can be presented individually.
(35) Data on congenital anomalies obtained from subjects receiving a vaccine should be compared with those obtained from an appropriately selected and documented control group(s) to assess background rates in non-exposed populations, and should be analyzed by study arm and dose where possible, e.g., in prospective clinical trials.

### 3.3 Data presentation

These guidelines represent a desirable standard for the presentation and publication of data on congenital anomalies following immunization to allow for comparability of data, and are recommended as an addition to data presented for the specific study question and setting. Additionally, it is recommended to refer to existing general guidelines for the presentation and publication of randomized controlled trials, systematic reviews, and meta-analyses of observational studies in epidemiology (e.g., statements of Consolidated Standards of Reporting Trials (CONSORT), of Improving the quality of reports of meta-analyses of randomized controlled trials (QUORUM), and of Meta-analysis Of Observational Studies in Epidemiology (MOOSE), respectively).

(36) All reported events of congenital anomalies should be presented according to the categories listed in guideline 31.
(37) Data on possible congenital anomalies events should be presented in accordance with data collection guidelines 1–24 and data analysis guidelines 31–34.
(38) Terms to describe congenital anomalies such as “low-grade”, “mild”, “moderate”, “high”, “severe” or “significant” are highly subjective, prone to wide interpretation, and should be avoided, unless clearly defined.
(39) Data should be presented with numerator and denominator (n/N) (and not only in percentages), if available.

Although immunization safety surveillance systems denominator data are usually not readily available, attempts should be made to identify approximate denominators. The source of the denominator data should be reported and calculations of estimates be described (e.g., manufacturer data like total doses distributed, reporting through Ministry of Health, coverage/population based data, etc.).

(40) The incidence of cases in the study population should be presented and clearly identified as such in the text. It would be useful to compare rates to background rates for these conditions. Useful resources include: EUROCAT prevalence tables (http://www.eurocat-network.eu/accessprevalencedata/prevalencetables). This allows that both exposed an unexposed cohorts can be compared with an external standard.
(41) If the distribution of data is skewed, median and range are usually the more appropriate statistical descriptors than a mean. However, the mean and standard deviation should also be provided.
(42) We recommend the studies powered to evaluate composite congenital anomaly outcomes also publish, or make data available that contains a description of individual anomalies. This will potentially allow meta-analysis of more discrete outcomes and help define the risk of less frequent anomalies.
(43) Any publication of data on congenital anomalies should include a detailed description of the methods used for data collection and analysis as possible. It is essential to specify:

• The study design;
• The method, frequency and duration of monitoring for congenital anomalies;
• The trial profile, indicating participant flow during a study including drop-outs and withdrawals to indicate the size and nature of the respective groups under investigation;
• The type of surveillance (e.g., passive or active surveillance);
• The characteristics of the surveillance system (e.g., population served, mode of report solicitation);
• The search strategy in surveillance databases;
• Comparison group(s), if used for analysis;
• The instrument of data collection (e.g., standardized questionnaire, diary card, report form);
• Whether the day of immunization was considered “day one” or “day zero” in the analysis;
• Whether the date of timing of first observation relative to vaccination^7^ and/or the date of first observation^3^ and/or the date of diagnosis^4^ was used for analysis; and
• Use of this case definition for congenital anomalies, in the abstract or methods section of a publication.^8^^,^^9^^,^^10^^,^^11^,^12^

## Figures and Tables

**Table 1:** List of organizations focused on research related to congenital anomalies, specific term and definitions used by each organization.

Organization	Congenital anomaly terminology	Definition
National Institute of Child Health and Human Development^a^	Birth defects	Birth defects are structural or functional abnormalities present at birth that can cause physical disability, intellectual and developmental disability (IDD), and other health problems. Some may be fatal, especially if not detected and treated early.
WHO^b^	Congenital Anomaly	Structural or functional anomalies that occur during intrauterine life and can be identified prenatally, at birth or later in life.
National Birth Defects Prevention Network^c^	Birth defects	Major anomaly – congenital abnormality that requires medical or surgical treatment, has a serious adverse effect on health and development, or has significant cosmetic impact Minor anomaly – congenital abnormality that does not require medical or surgical treatment, does not seriously affect health and development, and does not have significant cosmetic impact
Metropolitan Atlanta Congenital Defects Program^d^	Birth defects	Major structural or genetic birth defects as conditions that (1) result from a malformation, deformation, or disruption in one or more parts of the body, a chromosomal abnormality, or a known clinical syndrome; (2) are present at birth; and (3) have a serious, adverse effect on health, development, or functional ability.
March of Dimes^e^	Birth defects	Health conditions present at birth that change the shape or function of one or more parts of the body. Birth defects can cause problems in overall health, how the body develops or how the body works.
WHO/CDC/International Clearinghouse for Birth Defects Monitoring Systems^f^	Birth defects	Wide range of abnormalities of body structure or function that are present at birth and are of prenatal origin.

a https://www.nichd.nih.gov/health/topics/birthdefects/Pages/default.aspx.

b http://www.who.int/mediacentre/factsheets/fs370/en/.

c http://www.nbdpn.org/docs/SGSC_-_Ch3_Case_Definition_-_final_draft_3-24-15.pdf.

d http://www.cdc.gov/ncbddd/birthdefects/macdp.html.

e http://www.marchofdimes.org/.

f http://www.icbdsr.org/page.asp?p=9895&l=1.

## Footnotes

2 If the reporting center is different from the vaccinating center, appropriate and timely communication of the adverse event should occur.

3 The date and/or time of first observation of the first sign or symptom indicative for Congenital Anomalies can be used if date/time of onset is not known.

4 The date of diagnosis of an episode is the day post immunization when the event met the case definition at any level.

5 E.g., recovery to pre-immunization health status, spontaneous resolution, therapeutic intervention, persistence of the event, sequelae, death.

6 To determine the appropriate category, the user should first establish, whether a reported event meets the criteria for the lowest applicable level of diagnostic certainty, e.g., Level three. If the lowest applicable level of diagnostic certainty of the definition is met, and there is evidence that the criteria of the next higher level of diagnostic certainty are met, the event should be classified in the next category. This approach should be continued until the highest level of diagnostic certainty for a given event could be determined. Major criteria can be used to satisfy the requirement of minor criteria. If the lowest level of the case definition is not met, it should be ruled out that any of the higher levels of diagnostic certainty are met and the event should be classified in additional categories four or five.

7 The date and/or time of onset is defined as the time post immunization, when the first sign or symptom indicative for a Congenital Anomaly is clinically recognized. This may only be possible to determine in retrospect.

8 Use of this document should preferably be referenced by referring to the respective link on the Brighton Collaboration website (http://www.brightoncollaboration.org).

9 The end of an episode is defined as the time the event no longer meets the case definition at the lowest level of the definition.

10 An AEFI is defined as serious by international standards if it meets one or more of the following criteria: (1) it results in death, (2) is life-threatening, (3) it requires inpatient hospitalization or results in prolongation of existing hospitalization, (4) results in persistent or significant disability/incapacity, (5) is a congenital anomaly/birth defect, (6) is a medically important event or reaction.

11 If the evidence available for an event is insufficient because information is missing, such an event should be categorized as “Reported Congenital Anomalies with insufficient evidence to meet the case definition”.

12 An event does not meet the case definition if investigation reveals a negative finding of a necessary criterion (necessary condition) for diagnosis. Such an event should be rejected and classified as “Not a case of Congenital Anomalies”.

## References

[1] Khoury M.J. (1989). Epidemiology of birth defects. Epidemiol Rev.

[2] Naghavi M., Wang H., Lozano R., Davis A., Liang X., Zhou M. (2015). Global, regional, and national age-sex specific all-cause and cause-specific mortality for 240 causes of death, 1990–2013: a systematic analysis for the Global Burden of Disease Study 2013. Lancet.

[3] Rasmussen S.A., Olney R.S., Holmes L.B., Lin A.E., Keppler-Noreuil K.M., Moore C.A. (2003). Guidelines for case classification for the National Birth Defects Prevention Study. Birth Defects Res A Clin Mol Teratol.

[4] Wellesley D., Boyd P., Dolk H., Pattenden S. (2005). An aetiological classification of birth defects for epidemiological research. J Med Genet.

[5] (2011). Prevention and control of influenza with vaccines: recommendations of the Advisory Committee on Immunization Practices (ACIP), 2011. MMWR Morb Mortal Wkly Rep.

[6] (2012). Vaccines against influenza WHO position paper – November 2012. Wkly Epidemiol Rec.

[7] Christian L.M., Iams J.D., Porter K., Glaser R. (2011). Inflammatory responses to trivalent influenza virus vaccine among pregnant women. Vaccine.

[8] Christian L.M., Porter K., Karlsson E., Schultz-Cherry S., Iams J.D. (2013). Serum proinflammatory cytokine responses to influenza virus vaccine among women during pregnancy versus non-pregnancy. Am J Reprod Immunol.

[9] Chambers C.D., Johnson D., Xu R., Luo Y., Louik C., Mitchell A.A. (2013). Risks and safety of pandemic H1N1 influenza vaccine in pregnancy: birth defects, spontaneous abortion, preterm delivery, and small for gestational age infants. Vaccine.

[10] Deinard A.S., Ogburn P. (1981). A/NJ/8/76 influenza vaccination program: effects on maternal health and pregnancy outcome. Am J Obstet Gynecol.

[11] Heikkinen T., Young J., van Beek E., Franke H., Verstraeten T., Weil J.G. (2012). Safety of MF59-adjuvanted A/H1N1 influenza vaccine in pregnancy: a comparative cohort study. Am J Obstet Gynecol.

[12] Kallen B., Olausson P.O. (2012). Vaccination against H1N1 influenza with Pandemrix((R)) during pregnancy and delivery outcome: a Swedish register study. BJOG.

[13] Launay O., Krivine A., Charlier C., Truster V., Tsatsaris V., Lepercq J. (2012). Low rate of pandemic A/H1N1 2009 influenza infection and lack of severe complication of vaccination in pregnant women: a prospective cohort study. PLoS ONE.

[14] Louik C., Ahrens K., Kerr S., Pyo J., Chambers C., Jones K.L. (2013). Risks and safety of pandemic H1N1 influenza vaccine in pregnancy: exposure prevalence, preterm delivery, and specific birth defects. Vaccine.

[15] Mackenzie I.S., MacDonald T.M., Shakir S., Dryburgh M., Mantay B.J., McDonnell P. (2012). Influenza H1N1 (swine flu) vaccination: a safety surveillance feasibility study using self-reporting of serious adverse events and pregnancy outcomes. Br J Clin Pharmacol.

[16] Oppermann M., Fritzsche J., Weber-Schoendorfer C., Keller-Stanislawski B., Allignol A., Meister R. (2012). A(H1N1)v2009: a controlled observational prospective cohort study on vaccine safety in pregnancy. Vaccine.

[17] Pasternak B., Svanstrom H., Molgaard-Nielsen D., Krause T.G., Emborg H.D., Melbye M. (2012). Risk of adverse fetal outcomes following administration of a pandemic influenza A(H1N1) vaccine during pregnancy. JAMA.

[18] Rubinstein F., Micone P., Bonotti A., Wainer V., Schwarcz A., Augustovski F. (2013). Influenza A/H1N1 MF59 adjuvanted vaccine in pregnant women and adverse perinatal outcomes: multicentre study. BMJ.

[19] Sheffield J.S., Greer L.G., Rogers V.L., Roberts S.W., Lytle H., McIntire D.D. (2012). Effect of influenza vaccination in the first trimester of pregnancy. Obstet Gynecol.

[20] Munoz F.M., Englund J.A., Cheesman C.C., Maccato M.L., Pinell P.M., Nahm M.H. (2001). Maternal immunization with pneumococcal polysaccharide vaccine in the third trimester of gestation. Vaccine.

[21] McMillan M., Porritt K., Kralik D., Costi L., Marshall H. (2015). Influenza vaccination during pregnancy: a systematic review of fetal death, spontaneous abortion, and congenital malformation safety outcomes. Vaccine.

[22] Demicheli V., Jefferson T., Al-Ansary Lubna A., Ferroni E., Rivetti A., Di Pietrantonj C. (2014). Vaccines for preventing influenza in healthy adults. Cochrane database of systematic reviews.

[23] Fabiani M., Bella A., Rota M.C., Clagnan E., Gallo T., D’Amato M. (2015). A/H1N1 pandemic influenza vaccination: a retrospective evaluation of adverse maternal, fetal and neonatal outcomes in a cohort of pregnant women in Italy. Vaccine.

[24] Trotta F., Da Cas R., Spila Alegiani S., Gramegna M., Venegoni M., Zocchetti C. (2014). Evaluation of safety of A/H1N1 pandemic vaccination during pregnancy: cohort study. BMJ.

[25] Huang W.T., Tang F.W., Yang S.E., Chih Y.C., Chuang J.H. (2014). Safety of inactivated monovalent pandemic (H1N1) 2009 vaccination during pregnancy: a population-based study in Taiwan. Vaccine.

[26] (2015). Pertussis vaccines: WHO position paper – September 2015. Wkly Epidemiol Rec.

[27] (2013). Updated recommendations for use of tetanus toxoid, reduced diphtheria toxoid, and acellular pertussis vaccine (Tdap) in pregnant women—Advisory Committee on Immunization Practices (ACIP) 2012. MMWR Morb Mortal Wkly Rep.

[28] Munoz F.M., Bond N.H., Maccato M., Pinell P., Hammill H.A., Swamy G.K. (2014). Safety and immunogenicity of tetanus diphtheria and acellular pertussis (Tdap) immunization during pregnancy in mothers and infants: a randomized clinical trial. JAMA.

[29] Shakib J.H., Korgenski K., Sheng X., Varner M.W., Pavia A.T., Byington C.L. (2013). Tetanus, diphtheria, acellular pertussis vaccine during pregnancy: pregnancy and infant health outcomes. J Pediatr.

[30] Morgan J.L., Baggari S.R., McIntire D.D., Sheffield J.S. (2015). Pregnancy outcomes after antepartum tetanus, diphtheria, and acellular pertussis vaccination. Obstet Gynecol.

[31] Zheteyeva Y.A., Moro P.L., Tepper N.K., Rasmussen S.A., Barash F.E., Revzina N.V. (2012). Adverse event reports after tetanus toxoid, reduced diphtheria toxoid, and acellular pertussis vaccines in pregnant women. Am J Obstet Gynecol.

[32] (2006). Tetanus vaccine. Wkly Epidemiol Rec.

[33] Heinonen O., Slone D., Shapiro S. (1977). Birth defects and drugs in pregnancy.

[34] Czeizel A.E., Rockenbauer M. (1999). Tetanus toxoid and congenital abnormalities. Int J Gynaecol Obstet.

[35] Silveira C.M., Caceres V.M., Dutra M.G., Lopes-Camelo J., Castilla E.E. (1995). Safety of tetanus toxoid in pregnant women: a hospital-based case-control study of congenital anomalies. Bull World Health Organ.

[36] (1990). TORCH syndrome and TORCH screening. Lancet.

[37] Keller-Stanislawski B., Englund J.A., Kang G., Mangtani P., Neuzil K., Nohynek H. (2014). Safety of immunization during pregnancy: a review of the evidence of selected inactivated and live attenuated vaccines. Vaccine.

[38] Bar-Oz B., Levichek Z., Moretti M.E., Mah C., Andreou S., Koren G. (2004). Pregnancy outcome following rubella vaccination: a prospective controlled study. Am J Med Genet Part A.

[39] da Silva e Sa G.R., Camacho L.A., Stavola M.S., Lemos X.R., Basilio de Oliveira C.A., Siqueira M.M. (2011). Pregnancy outcomes following rubella vaccination: a prospective study in the state of Rio de Janeiro Brazil, 2001–2002. J Infect Dis.

[40] Badilla X., Morice A., Avila-Aguero M.L., Saenz E., Cerda I., Reef S. (2007). Fetal risk associated with rubella vaccination during pregnancy. Pediatr Infect Dis J.

[41] Minussi L., Mohrdieck R., Bercini M., Ranieri T., Sanseverino M.T., Momino W. (2008). Prospective evaluation of pregnant women vaccinated against rubella in southern Brazil. Reprod Toxicol.

[42] Pardon F., Vilarino M., Barbero P., Garcia G., Outon E., Gil C. (2011). Rubella vaccination of unknowingly pregnant women during 2006 mass campaign in Argentina. J Infect Dis.

[43] Soares R.C., Siqueira M.M., Toscano C.M., Maia Mde L., Flannery B., Sato H.K. (2011). Follow-up study of unknowingly pregnant women vaccinated against rubella in Brazil, 2001–2002. J Infect Dis.

[44] Hamkar R., Jalilvand S., Abdolbaghi M.H., Esteghamati A.R., Hagh-Goo A., Jelyani K.N. (2006). Inadvertent rubella vaccination of pregnant women: evaluation of possible transplacental infection with rubella vaccine. Vaccine.

[45] Castillo-Solorzano C., Reef S.E., Morice A., Vascones N., Chevez A.E., Castalia-Soares R. (2011). Rubella vaccination of unknowingly pregnant women during mass campaigns for rubella and congenital rubella syndrome elimination, the Americas 2001–2008. J Infect Dis.

[46] Sukumaran L., McNeil M.M., Moro P.L., Lewis P.W., Winiecki S.K., Shimabukuro T.T. (2015). Adverse events following measles, mumps, and rubella vaccine in adults reported to the vaccine adverse event reporting system (VAERS), 2003–2013. Clin Infect Dis.

[47] Burton A.E., Robinson E.T., Harper W.F., Bell E.J., Boyd J.F. (1984). Fetal damage after accidental polio vaccination of an immune mother. J R Coll Gen Pract.

[48] Harjulehto-Mervaala T., Aro T., Hiilesmaa V.K., Hovi T., Saxen H., Saxen L. (1994). Oral polio vaccination during pregnancy: lack of impact on fetal development and perinatal outcome. Clin Infect Dis.

[49] Suzano C.E., Amaral E., Sato H.K., Papaiordanou P.M., Campinas Group on Yellow Fever Immunization during Pregnancy (2006). The effects of yellow fever immunization (17DD) inadvertently used in early pregnancy during a mass campaign in Brazil. Vaccine.

[50] Cavalcanti D.P., Salomao M.A., Lopez-Camelo J., Pessoto M.A., Campinas Group of Yellow Fever Immunization during Pregnancy (2007). Early exposure to yellow fever vaccine during pregnancy. Trop Med Int Health.

[51] Wilson E., Goss M.A., Marin M., Shields K.E., Seward J.F., Rasmussen S.A. (2008). Varicella vaccine exposure during pregnancy: data from 10 years of the pregnancy registry. J Infect Dis.

[52] Angelo M.G., David M.P., Zima J., Baril L., Dubin G., Arellano F. (2014). Pooled analysis of large and long-term safety data from the human papillomavirus-16/18-AS04-adjuvanted vaccine clinical trial programme. Pharmacoepidemiol Drug Saf.

[53] Garland S.M., Ault K.A., Gall S.A., Paavonen J., Sings H.L., Ciprero K.L. (2009). Pregnancy and infant outcomes in the clinical trials of a human papillomavirus type 6/11/16/18 vaccine: a combined analysis of five randomized controlled trials. Obstet Gynecol.

[54] Dana A., Buchanan K.M., Goss M.A., Seminack M.M., Shields K.E., Korn S. (2009). Pregnancy outcomes from the pregnancy registry of a human papillomavirus type 6/11/16/18 vaccine. Obstet Gynecol.

[55] Goss M.A., Lievano F., Buchanan K.M., Seminack M.M., Cunningham M.L., Dana A. (2015). Final report on exposure during pregnancy from a pregnancy registry for quadrivalent human papillomavirus vaccine. Vaccine.

[56] Angelo M.G., Zima J., Tavares Da Silva F., Baril L., Arellano F. (2014). Post-licensure safety surveillance for human papillomavirus-16/18-AS04-adjuvanted vaccine: more than 4 years of experience. Pharmacoepidemiol Drug Saf.

[57] Moro P.L., Zheteyeva Y., Lewis P., Shi J., Yue X., Museru O.I. (2015). Safety of quadrivalent human papillomavirus vaccine (Gardasil) in pregnancy: adverse events among non-manufacturer reports in the Vaccine Adverse Event Reporting System, 2006–2013. Vaccine.

[58] Nakalembe M., Mirembe F.M., Banura C. (2015). Vaccines against human papillomavirus in low and middle income countries: a review of safety, immunogenicity and efficacy. Infect Agents Cancer.

[59] Joura E.A., Giuliano A.R., Iversen O.E., Bouchard C., Mao C., Mehlsen J. (2015). A 9-valent HPV vaccine against infection and intraepithelial neoplasia in women. N Engl J Med.

[60] Makris M.C., Polyzos K.A., Mavros M.N., Athanasiou S., Rafailidis P.I., Falagas M.E. (2012). Safety of hepatitis B, pneumococcal polysaccharide and meningococcal polysaccharide vaccines in pregnancy: a systematic review. Drug Saf.

[61] Zheteyeva Y., Moro P.L., Yue X., Broder K. (2013). Safety of meningococcal polysaccharide-protein conjugate vaccine in pregnancy: a review of the Vaccine Adverse Event Reporting System. Am J Obstet Gynecol.

[62] Ouandaogo C.R., Yameogo T.M., Diomande F.V., Sawadogo C., Ouedraogo B., Ouedraogo-Traore R. (2012). Adverse events following immunization during mass vaccination campaigns at first introduction of a meningococcal A conjugate vaccine in Burkina Faso, 2010. Vaccine.

[63] (2008). Update on overall prevalence of major birth defects—Atlanta, Georgia, 1978–2005. MMWR Morb Mortal Wkly Rep.

[64] (2009). Population-based Birth Defects Surveillance data from selected states, 2002–2006. Birth Defects Res A Clin Mol Teratol.

[65] Kohl K.S., Gidudu J., Bonhoeffer J., Braun M.M., Buettcher M., Chen R.T. (2007). The development of standardized case definitions and guidelines for adverse events following immunization. Vaccine.

[66] Obican S., Scialli A.R. (2011). Teratogenic exposures. Am J Med Genet Part C: Semin Med Genet.

[67] Shiota K. (2009). Variability in human embryonic development and its implications for the susceptibility to environmental teratogenesis. Birth Defects Res A Clin Mol Teratol.

[68] Rasmussen S.A. (2012). Human teratogens update 2011: can we ensure safety during pregnancy?. Birth Defects Res A Clin Mol Teratol.

[69] International Conference on Harmonization of Technical Requirements for Registration of Pharmaceuticals for Human Use. 10.4103/0976-500X.162004 PMC4544148 26312010

[70] Council for International Organizations of Medical Sciences.

