# Supplementary material for: Congenital anomalies: Case definition and guidelines for data collection, analysis, and presentation of immunization safety data
Source: Vaccine. 2016 Dec 1;34(49):6015–26. doi: 10.1016/j.vaccine.2016.03.047 (PMC5139892; doi:10.1016/j.vaccine.2016.03.047)
Supplement: Supplementary file 1 [file mmc1.docx]

**Appendix A**

**Table**: Categories of major external structural congenital anomalies listed by several commonly referenced birth defects registries.

|  | **National Birth Defects Prevention Network** | **South Australian Birth Defects Register** | **Public Health Agency of Canada** | **Eurocat** | **WHO/CDC/ International Clearinghouse for Birth Defects Monitoring Systems** | **Metropolitan Atlanta Congenital Defects Program*** |
| --- | --- | --- | --- | --- | --- | --- |
| Stillbirths |  |  | X |  |  |  |
| Conjoined twins |  |  |  | X |  | X |
| **Central nervous system anomalies** |  |  |  |  |  |  |
| Neural tube defects |  | X | X | X |  |  |
| Anencephalus & similar anomalies | X | X | X | X | X | X |
| Spina bifida | X | X | X | X | X (open vs. closed) | X |
| Encephalocele | X | X | X | X | X | X |
| Microcephalus & brain reduction |  | X | X | X |  | X |
| Congenital hydrocephalus |  | X | X | X |  | X |
| Craniorachischisis |  |  |  |  | X | X |
| Iniencephaly |  |  |  |  | X | X |
| Other specified & unspecified CNS anomalies |  |  | X |  |  | X |
| Holoprosencephaly | X |  |  | X |  | X (listed under reduction deformities of brain) |
| **Eye anomalies** |  |  |  |  |  |  |
| Anophthalmos, microphthalmos | X |  | X | X |  | X |
| Congenital cataract | X |  |  | X |  | X |
| Congenital glaucoma |  |  |  | X |  |  |
| Other eye anomalies |  |  | X |  |  | X |
| **Ear face & neck anomalies** |  |  |  |  |  |  |
| Anotia/microtia | X | X |  | X |  | X |
| Anomalies of ear causing impairment |  |  | X |  |  | X |
| Other ear anomalies |  |  | X |  |  | X |
| Anomalies of face & neck |  |  | X |  |  | X |
| **Respiratory system anomalies** |  |  |  |  |  |  |
| Nose anomalies |  |  | X |  |  | X |
| Other respiratory system anomalies |  |  | X |  |  | X |
| Choanal atresia | X |  |  |  |  | X |
| Cleft lip and palate | X | X | X |  | X | X |
| Cleft palate alone (without cleft lip) | X | X | X |  | X | X |
| Cleft lip alone (without cleft palate) | X | X | X |  | X | X |
| Cleft palate with cleft lip |  |  | X |  |  | X |
| **Genital organ anomalies** |  |  |  |  |  |  |
| Hypospadias, epispadias | X | X | X | X | X | X |
| Indeterminate sex |  |  |  | X |  | X |
| Other genital organ anomalies |  |  | X |  |  | X |
| **Urinary system anomalies** |  |  |  |  |  |  |
| Bladder exstrophy | X |  |  | X |  | X |
| Cloacal exstrophy | X |  |  |  |  | X (listed under other anomalies of intestine) |
| Other urinary system anomalies |  |  | X |  |  | X |
| **Musculoskeletal anomalies** |  |  |  |  |  |  |
| Certain musculoskeletal anomalies |  |  | X |  |  |  |
| Clubfoot (talipes equinovarus) | X | X | X | X | X | X (listed under varus [inward] deformities of feet) |
| Polydactyly, syndactyly |  | X | X | X |  | X |
| Craniosynostosis | X |  |  | X |  | X (listed under anomalies of skull and face bones) |
| Gastroschisis | X | X |  | X | X | X (listed under anomalies of abdominal wall) |
| Omphalocele | X |  |  | X | X | X (listed under anomalies of abdominal wall) |
| Exomphalos |  | X |  |  | X | X (listed under anomalies of abdominal wall) |
| Limb reduction anomalies | X | X | X | X | X | X |
| Skeletal dysplasia |  |  |  | X |  |  |
| Congenital absence of finger(s) |  |  |  |  | X | X (listed under reduction defects of upper limb) |
| Other, unspecified limb anomalies |  |  | X |  |  | X |
| Congenital constriction bands/amniotic band |  |  |  | X |  | X |
| Anomalies of abdominal wall |  |  | X | X |  | X |
| Other musculoskeletal anomalies |  |  | X |  |  | X |
| Anomalies of integument |  |  | X |  |  | X |
| Other selected |  |  |  |  |  |  |
| Congenital syphilis syndrome |  | X |  |  |  | X |
| Congenital rubella syndrome |  | X |  |  |  | X |
| Fetal alcohol syndrome |  | X |  | X |  | X |
| Non-immune fetal hydrops |  | X |  |  |  |  |
| Haemangioma |  | X |  |  |  | X |
| Lymphangioma |  | X |  |  |  | X |
| Congenital skin disorders |  |  |  | X |  | X |
| Other & unspecified anomalies |  |  | X |  |  | X |
| Lateral anomalies |  |  |  | X |  |  |
| Teratogenic syndromes with malformations |  |  |  | X |  |  |

*Due to space constraints, not all congenital anomalies listed by the Metropolitan Atlanta Congenital Defects Program are included in this table. Please visit their website and refer to the MACDP 6-digit Code Defect List (http://www.cdc.gov/ncbddd/birthdefects/documents/macdpcode0807.pdf)

**Appendix B**

**Table**: Categories of major internal structural congenital anomalies listed by several commonly referenced birth defects registries.

|  | **National Birth Defects Prevention Network** | **South Australian Birth Defects Register** | **Public Health Agency of Canada** | **Eurocat** | **WHO/CDC/ International Clearinghouse for Birth Defects Monitoring Systems** | **Metropolitan Atlanta Congenital Defects Program*** |
| --- | --- | --- | --- | --- | --- | --- |
| **Congenital heart defects** |  |  |  |  |  |  |
| Aortic valve stenosis | X |  |  |  |  | X |
| Common truncus (truncus arteriosus or TA) | X |  | X | X |  | X |
| Transposition of great vessels | X | X | X | X |  | X |
| Tetralogy of Fallot | X | X | X | X |  | X |
| Interrupted aortic arch (IAA) | X |  |  | X |  | X (listed under other anomalies of aorta) |
| Double outlet right ventricle (DORV) | X |  |  | X |  | X (listed under transposition of the great vessels) |
| Common ventricle (Single ventricle) | X |  | X | X |  | X |
| Ventricular septal defect | X | X | X | X |  | X |
| Atrial septal defect | X | X | X | X |  | X |
| Atrioventricular septal defect (Endocardial cushion defects) | X |  | X | X |  | X |
| Tricuspid valve atresia and stenosis | X |  |  | X |  | X |
| Mitral valve anomalies |  |  |  | X |  | X |
| Other septal closure defects |  |  | X |  |  | X |
| Heart valve anomalies |  |  | X |  |  | X |
| Pulmonary valve atresia and stenosis | X |  |  | X (separated) |  | X |
| Hypoplastic left heart syndrome | X | X | X | X |  | X |
| Hypoplastic right heart |  |  |  | X |  | X (listed under other specified anomalies of the heart) |
| Ebstein anomaly | X |  |  | X |  | X |
| Other heart anomalies |  |  | X |  |  | X |
| Circulatory system anomalies |  |  | X |  |  | X |
| Coarctation of aorta | X | X | X | X |  | X |
| Patent ductus arteriosus |  | X |  |  |  | X (listed under other congenital anomalies of circulatory system |
| Other anomalies of aorta |  |  | X |  |  | X |
| Total anomalous pulmonary venous connection (TAPVC) | X |  |  | X |  | X (listed under Anomalies of Great Veins) |
| Pulmonary artery anomalies |  |  | X |  |  | X |
| Patent Ductus Arteriosus as only CHD in term infants (>=37 weeks) |  |  |  | X |  | X (listed under other congenital anomalies of circulatory system |
| Situs inversus |  |  |  | X |  | X (listed under other specified anomalies of the heart) |
| Other circulatory system anomalies |  |  | X |  |  | X |
| **Respiratory system anomalies** |  |  |  |  |  |  |
| Nose anomalies |  |  | X |  |  | X |
| Lung agenesis & hypoplasia |  | X | X |  |  | X |
| Cystic adenomatous malformation of lung |  |  |  | X |  | X |
| Other respiratory system anomalies |  |  | X |  |  | X |
| Choanal atresia | X |  |  | X |  | X |
| Cleft lip and palate | X | X | X | X (w or w/o palate) |  | X |
| Cleft palate alone (without cleft lip) | X | X | X | X |  | X |
| Cleft lip alone (without cleft palate) | X | X | X |  |  | X |
| Cleft palate with cleft lip |  |  | X |  |  | X |
| **Digestive system anomalies** |  |  |  |  |  |  |
| T-E fistula, esophageal atresia & stenosis | X | X | X | X |  | X |
| Biliary atresia | X |  |  |  |  | X (listed under anomalies of gallbladder, bile ducts, and liver) |
| Other upper alimentary tract anomalies |  |  | X |  |  | X |
| Intestinal, anorectal atresia & stenosis |  |  | X | X |  | X |
| - rectal and large intestinal atresia/stenosis | X | X |  |  |  | X |
| - duodenal atresia or stenosis |  |  |  | X |  | X (listed under atresia and stenosis of small intestine) |
| - small intestinal atresia/stenosis | X |  |  | X |  | X (listed under atresia and stenosis of small intestine) |
| Pyloric stenosis |  | X |  |  |  | X |
| Hirschsprung disease |  | X |  | X |  | X |
| Atresia of bile ducts |  |  |  | X |  | X (listed under anomalies of gallbladder, bile ducts, and liver) |
| Annular pancreas |  |  |  | X |  | X (listed under anomalies of pancreas) |
| Other digestive system anomalies |  |  | X |  |  | X |
| **Urinary system anomalies** |  |  |  |  |  |  |
| Renal agenesis/hypoplasia & dysgenesis | X | X | X | X(bilateral renal agenesis including Potter syndrome) |  | X |
| Congenital posterior urethral valves | X |  |  | X (and/or prune belly) |  | X (listed under atresia and stenosis of urethra and bladder neck) |
| Undescended testicle |  | X |  |  |  | X |
| Vesico-ureteric reflex |  | X |  |  |  | X (listed under other specified anomalies of ureter) |
| Cystic kidney disease |  |  | X |  |  | X |
| Multicystic renal dysplasia |  |  |  | X |  | X (listed under cystic kidney disease) |
| Congenital hydronephrosis |  |  |  | X |  | X (listed under obstructive defects of renal pelvis and ureter) |
| Other urinary system anomalies |  |  | X |  |  | X |
| **Musculoskeletal anomalies** |  |  | X |  |  |  |
| Certain musculoskeletal anomalies |  |  | X |  |  | X |
| Congenital dislocation of hip/developmental dysplasia of hip |  | X | X | X |  |  |
| Diaphragmatic hernia | X | X |  | X |  | X (listed under anomalies of diaphragm) |
| Anomalies of integument |  |  | X |  |  |  |
| **Other selected congenital anomalies** |  |  |  |  |  |  |
| Congenital syphilis syndrome |  | X |  |  |  | X |
| Congenital rubella syndrome |  | X |  |  |  | X |
| Maternal infections resulting in malformations |  |  |  | X |  |  |
| Fetal alcohol syndrome |  | X |  | X |  | X |
| Valproate syndrome |  |  |  | X |  |  |
| Non-immune fetal hydrops |  | X |  |  |  |  |
| Haemangioma |  | X |  |  |  | X |
| Lymphangioma |  | X |  |  |  | X |
| Vascular disruption anomalies |  |  |  | X |  |  |
| VATER/VACTERL |  |  |  | X |  |  |
| Other & unspecified anomalies |  |  | X |  |  | X |

*Due to space constraints, not all congenital anomlies listed by the ___ are included in this table. Please visit their website and refer to the MACDP 6-digit Code Defect List (http://www.cdc.gov/ncbddd/birthdefects/documents/macdpcode0807.pdf)

**Appendix C**

**Table**: Categories of major functional congenital anomalies listed by several commonly referenced birth defects registries.

|  | **National Birth Defects Prevention Network** | **South Australian Birth Defects Register** | **Public Health Agency of Canada** | **Eurocat** | **WHO/CDC/ International Clearinghouse for Birth Defects Monitoring Systems** | **Metropolitan Atlanta Congenital Defects Program*** |
| --- | --- | --- | --- | --- | --- | --- |
| Down syndrome | X | X | X | X |  | X |
| Other chromosomal anomalies |  |  | X |  |  | X |
| Trisomy 13 | X | X | X | X |  | X (listed under Patau syndrome) |
| Trisomy 18 | X | X | X | X |  | X (listed under Edwards syndrome) |
| Deletion 22 q11 | X |  |  |  |  |  |
| Autosomal syndromes |  |  | X |  |  | X (listed under other conditions due to autosomal anomalies) |
| Sex chromosome conditions |  |  | X |  |  | X (listed under other conditions due to sex chromosome anomalies) |
| Turner syndrome | X | X |  | X |  | X (listed under gonadal dysgenesis) |
| Klinefelter syndrome |  |  |  | X |  | X |
| Achondroplasia |  | X |  |  |  | X (listed under chondrodystrophy) |
| Osteogenesis imperfecta |  | X |  |  |  | X (listed under osteodystrophies) |
| Genetic syndromes + microdeletions |  |  |  | X |  |  |
| Other & unspecified anomalies |  |  | X |  |  | X |
| Congenital hypothyroidism |  | X |  |  |  | X |
| Phenylketonuria |  | X |  |  |  | X |
| Galactosaemia |  | X |  |  |  |  |
| Albinism |  | X |  |  |  | X |
| Cystic fibrosis |  | X |  |  |  | X |
| Other metabolic disorders |  | X |  |  |  | X (multiple metabolic disorder listed) |
| **Hematological/Immune** |  |  |  |  |  |  |
| Haemolytic anaemias |  | X |  |  |  |  |
| Thalassaemias |  | X |  |  |  |  |
| Coagulation defects |  | X |  |  |  |  |

*Due to space constraints, not all congenital anomalies listed by the Metropolitan Atlanta Congenital Defects Program are included in this table. Please visit their website and refer to the MACDP 6-digit Code Defect List (http://www.cdc.gov/ncbddd/birthdefects/documents/macdpcode0807.pdf)
